# Complications after proximal abducting ulnar osteotomy and prognostic factors in 66 dogs

**DOI:** 10.1111/vsu.13697

**Published:** 2021-08-09

**Authors:** Alan Danielski, Alexander Krekis, Russell Yeadon, Miguel Angel Solano, Tim Parkin, Aldo Vezzoni, Ingo Pfeil

**Affiliations:** ^1^ The Ralph Veterinary Referral Centre Marlow UK; ^2^ Department of Veterinary Medicine and Animal Sciences University of Naples “Federico II” Naples Italy; ^3^ Davies Veterinary Specialists Higham Gobion UK; ^4^ Lumbry Park Alton UK; ^5^ Fitzpatrick Referrals Godalming UK; ^6^ Bristol Veterinary School University of Bristol Langford UK; ^7^ Clinica Veterinaria Vezzoni Cremona Italy; ^8^ Tierärztliche Klinik Dresden Germany

## Abstract

**Objective:**

To report complications and prognostic factors in dogs undergoing proximal abducting ulnar osteotomy (PAUL). To evaluate the ability to predict complications on the basis of post‐operative radiographic examination.

**Study Design:**

Retrospective cohort study.

**Animals:**

Sixty‐six dogs.

**Methods:**

Medical records of dogs treated with PAUL between 2014 and 2019 were reviewed for demographics, intraoperative findings, and post‐operative complications. Post‐operative radiographs were reviewed by two masked expert orthopedic surgeons, who were asked to predict the likelihood of major mechanical complications. The prognostic value of variables was tested with univariate and multivariable logistic regression. Inter‐investigator agreement to predict complications was evaluated with two‐by‐two tables and kappa coefficient.

**Results:**

Seventy‐four PAULs in 66 dogs were included. Duration of follow‐up ranged from 12 to 75 months (median: 53 months). Post‐operative complications were documented in 19/74 limbs (16 dogs), including major complications in 13 limbs. These complications consisted mainly of non‐union (six limbs), implant failure (two limbs), and infection (two limbs) requiring revision surgery in nine limbs. Body weight was the only variable associated with an increased risk of post‐operative complications (*p* = .04). Agreement between expert predictions was low (respectively *k* = −0.08 and *k* = 0.11).

**Conclusion:**

Major complications were reported in one fourth of limbs treated with PAUL and were more likely as body weight increased. Suboptimal plate and screw placement or osteotomy reduction on post‐operative radiographs were poorly predictive of complications.

**Clinical Significance:**

Complications are fairly common after PAUL, particularly in heavier dogs, and post‐operative radiographic examination seems unreliable to predict those.

## 1 INTRODUCTION

Canine elbow dysplasia is a debilitating developmental elbow disease affecting mainly young large and giant dog breeds.^1^ Elbow incongruency is thought to be an etiopathogenic factor in the development of elbow dysplasia.^1^ Medial compartment disease is one potential manifestation of elbow dysplasia and commonly involves erosion of the cartilage of the medial humeral condyle and of the medial coronoid process.^2^, ^3^ The degree of cartilage damage may vary from focal disease to deep longitudinal abrasion of cartilage and subchondral bone to full‐thickness cartilage damage and bone eburnation.^1^, ^4^

Described treatment options for management of medial compartment disease include the use of systemic analgesic medications, physiotherapy, hydrotherapy, intra‐articular injections, load‐shifting modifying osteotomies, and partial or total joint replacement.^4^, ^5^, ^6^, ^7^ Among the load‐shifting modifying osteotomy techniques, proximal abducting ulnar osteotomy (PAUL) has been reported to be a potentially effective option to treat elbow pain associated with medial compartment disease.^8^, ^9^

PAUL involves a transverse ulnar osteotomy secured with a specific locking plate (Advanced Locking Plate System [ALPS] PAUL, KYON Veterinary Surgical Products, Boston, Massachusetts) such that mild abduction of the ulna results.^8^ According to the manufacturer, the resulting corrective limb alignment is aimed at unloading the medial joint compartment of the elbow, alleviating lameness, stiffness, and joint pain.^8^ However, a recent ex vivo study seemed to demonstrate that the effect of the PAUL on the congruent elbow was limited to a decrease of intra‐articular contact area in both medial and lateral compartments whilst, in incongruent elbows, its effect was to possibly alleviate pressures in the medial compartment.^9^

Although this technique has been commercially available for several years, there have been few scientific reports regarding its clinical application and outcomes. The primary objective of this study is to retrospectively report complication rate and associated risk factors for development of complications in dogs that underwent PAUL during a 5‐year period. The secondary objective of this study was to prospectively evaluate whether evaluation of post‐operative radiographs by two highly experienced surgeons in PAUL, specifically assessing for technical errors or deviations away from recommended surgical technique, might help to predict subsequent development of post‐operative complications. Our null hypotheses regarding this second study objective were that post‐operative radiographic assessment by experienced surgeons in PAUL would not be predictive for development of complications, and that inter‐observer variability between experienced experts would be poor.

## 2 MATERIAL AND METHODS

Medical records (2014–2019) for all dogs treated by PAUL were reviewed. Criteria for inclusion were availability of clinical records (including referring veterinarian history) for at least 1 year from the surgery date and presence of immediate post‐operative and 6‐week follow‐up radiographs. Data collected included signalment, implant size, use of post‐operative bandaging, and presence and nature of any peri‐ and post‐operative complications. For those dogs that had follow‐up radiographs performed at the referring vet, radiographs and clinical history up to the time of writing of this manuscript were requested and assessed by an ECVS Boarded Surgeon. Dogs where follow‐up radiographs and clinical history were not available were excluded. Each PAUL was performed by one of four experienced ECVS Boarded Surgeons following the principles indicated by the manufacturer (http://www.kyon.ch/wp-content/uploads/2013/05/Vezzoni_PAUL-small.pdf).

Post‐operative care consisted of 6 weeks of cage confinement with three sessions of leash‐walking of not more than 5 min per session initially, progressing to not more than 20 min per session by week 6 post‐operatively. After 6 weeks, dogs were allowed to be confined to one room with a non‐slippery floor and lead‐only walking was encouraged until week 12. Any dog with residual lameness at 6 weeks was reassessed clinically and radiographically at 12 weeks or until lameness resolved. Complications were classified as previously described by Cook et al. into *minor* (not requiring additional surgical or medical treatment to resolve) or *major* (requiring surgical or medical treatment to resolve).^10^

All post‐operative radiographs were anonymized and were subsequently reviewed by two orthopedic surgeons (Author 6; AV and Author 7; IP, hereafter referred to as “Experts”) highly experienced in performing PAUL. Experts were unaware of clinical data including incidence of complications at the time of radiographic interpretation. These experts had numerous years of experience in performing this technique and they were involved in the development and teaching of it for at least 9 years. They were asked to answer “Yes” or “No” to the question “*Do you believe that these post‐operative radiographs show major technical errors that will lead to development of post‐operative complications*?” and, if this was the case, to specify the nature of the technical error. Technical errors were subcategorized into “*Implant errors*” and “*Reduction errors*” for statistical purposes.

“*Implant errors*” were subcategorized into errors relating to the plate (too cranial, too caudal, too oblique, too proximal, too distal or not adequately in contact with the ulna, inappropriate size), and errors relating to the screws (inappropriate length, inappropriate size, screws not perpendicular to the plate [and therefore not locked properly with the plate]). “*Reduction errors*” were subcategorized to include inappropriate “caudal kick” (defined as a caudo‐cranial step at level of the osteotomy due to caudal movement of the proximal ulnar segment in the sagittal plane), excessive gap at level of the osteotomy, osteotomy too proximal, and osteotomy too distal.

### 2.1 Statistics

Univariate and then multivariable logistic regression were used to identify variables potentially associated with the likelihood of complications. Initial screening of all variables was conducted and variables with an initial univariate *p*‐value less than .2 were available for potential inclusion in final multivariable models. Agreement between expert prediction of complications and between subcategories of expert‐identified technical errors with eventual complications was evaluated using two‐by‐two tables and calculation of kappa values.

Agreement among observers was calculated using multirater (Fleiss') kappa. Kappa values were interpreted such that *k*‐value of 0 would indicate no agreement beyond what was expected attributable to chance alone. The value of −1.00 would indicate total disagreement and +1.00 would represent perfect agreement. Further interpretation followed the guidelines outlined by Landis and Koch,^11^ where strength of the kappa coefficients is interpreted in the following manner: 0.01–0.20 slight; 0.21–0.40 fair; 0.41–0.60 moderate; 0.61–0.80 substantial; and 0.81–1.00 almost perfect.

## 3 RESULTS

Seventy‐four limbs treated by PAUL (66 dogs) met the inclusion criteria. Five dogs (five procedures) were excluded because of the lack of post‐operative history or follow‐up radiographs. A minimum 1‐year follow‐up assessment (based on up‐to‐date clinical history including documentation of physical examination provided by the referring veterinary surgeon and by our clinical records) was available for every dog included in the study (median 53 months, range 12–75 months post‐operatively at time of last physical examination).

Twenty‐five dogs were female (8 entire, 17 neutered) and 41 male (19 entire, 22 neutered). The breed most commonly represented was Labrador Retriever (*n* = 38, 57.5%) followed by crossbreed (*n* = 6) and Staffordshire Bull Terriers (*n* = 5). Mean body weight was 30.8 ± 8.68 kg. Median age at the time of surgery was 34.5 months (range 5–122 months). Eight dogs underwent bilateral staged PAULs (median 3 months in between surgeries, range 1–22 months).

Sixty‐six of 74 limbs had a post‐operative bandage applied whilst 8/74 did not have one. The bandage was maintained in situ for a median time of 5 days (range 0–21 days).

The plate size most commonly used was 10–3 mm (30/74 procedures, 40.5%) followed by 10–2 mm (*n* = 28/74, 37.8%), 8–2 mm (*n* = 9/74, 12.1%), 8–3 mm (*n* = 6/74, 8.1%), and 11–3 mm (*n* = 1/74, 1.3%).

Post‐operative complications were recorded in 19/74 limbs (16 dogs). Thirteen of 19 limbs had a major complication, as described in Table 1. Five of 13 limbs with major complications needed revision surgery to treat chronic instability following osteotomy viable non‐union (defined as an osteotomy that fails to progress to bony union regardless of healing time as assessed by two boarded surgeons), 1 limb needed revision surgery to treat delayed union, 2 limbs suffered ulnar fracture at level of the most distal screw (creating a segmental fracture that nullified the abducting effect of the PAUL plate), 2 limbs suffered breakage of at least one screw in the proximal osteotomy segment, 1 limb sustained severe infection that required plate removal, 1 limb had persistent pain on pressure application over the plate which required plate removal, and 1 limb sustained an infection that required medical treatment (Table 1). The two limbs that suffered ulnar fracture at the level of the most distal screw required no further intervention as bone healing progression at the osteotomy site was considered satisfactory at the time of the 6‐week follow‐up radiographs. The owners of both dogs declined to have further procedures performed. All six limbs with non‐union of the osteotomy had radiographic evidence of proximal screw breakage or radiolucency of the adjacent bone. Three of the 13 dogs that suffered major complications underwent bilateral staged surgery (the interval between surgeries was 3 months in one dog, 11 in the second, and 22 in the third). Nine PAUL (13%) plates were eventually removed (in five limbs because of non‐union, in one limb because of persistent pain, and in three limbs because of infection) at median 7 months (range 1.5–61 months) post‐operatively.

**TABLE 1:** Dogs that sustained major complications

Case	Breed	Age	Sex	Weight (kg)	Implant size	Complication	Time	Treatment
1	Labrador	4 y 4 m	F	20.3	8–3	First screw head broken, radiolucency around second screw	12 w	Osteotomy healed well, PAUL plate removed at 15 w
4	Crossbreed	4 y 11 m	M	24	10–2	Ulnar fracture through distal screw hole (loss of PAUL effect)	6 w	No treatment. Complete bone healing confirmed at 12 w
7	Estrela Mountain Dog	1 y 3 m	M	37	10–2	Radiolucency around first and third proximal screws, second screw loose with migration, delayed union at osteotomy site	5 w	PAUL plate removed, autogenous cancellous bone graft and 2.7 mm LCP plate applied
14	Old English Shepherd Dog	9 y 1 m	Mn	43	10–3	Ulnar fracture through distal screw hole (loss of PAUL effect)	6 w	No treatment. Satisfactory bone healing progression at osteotomy site was confirmed at 6 w
16	Springer Spaniel	8 y 8 m	M	27	8–3	Breakage of three proximal screws, hypertrophic viable non‐union of osteotomy	8 m	PAUL plate removed, autogenous cancellous bone graft applied
23	Labrador	3 y 8 m	M	36	10–3	Radiolucency around three proximal screws, hypertrophic viable non‐union of osteotomy	5 y	PAUL plate removed, autogenous cancellous bone graft and 3.5 mm LCP plate applied
35	Labrador	6 y	Fn	36	10–3	Radiolucency around three proximal screws, hypertrophic viable non‐union of osteotomy	8 m	PAUL plate removed, autogenous cancellous bone graft applied
36	Labrador	5 y 6 m	Mn	38	10–3	Pain on pressure over distal portion of plate	12 w	PAUL plate removed at 4 m
47	Mastiff cross	4 y 1 m	Fn	35	10–2	SSI (*Staphylococcus* spp. cultured). Radiolucency/reabsorption around three proximal screws	6 w	PAUL plate removed and type‐IA linear ESF applied
52	Rottweiler	3 y 11 m	Fn	32.4	10–2	First proximal screw broken, second screw loose with migration, hypertrophic viable non‐union, SSI (*Staphylococcus* spp. cultured)	16 w	Systemic antibiotic therapy, PAUL plate removed at 6 months
55	Labrador	3 y 11 m	M	28.3	10–2	Radiolucency around first and third proximal screws, second screw broken, SSI (suspected, no organism cultured)	12 w	PAUL plate removed at 4 months
69	Mastiff	8 y	Fn	45.3	11–3	SSI (suspected, no organism cultured)	2 w	Systemic antibiotic therapy for 2 weeks
70	German Shepherd Dog	1 y 10 m	M	48	10–3	Second screw loose with migration, radiolucency around first and third screws, oligotrophic viable non‐union	12 w	Three proximal screws replaced with 2.7 mm cortical screws (directed at different angles), autogenous cancellous bone graft and additional 3.5 mm LCP plate applied caudally

Abbreviations: ESF, external skeletal fixator; F, female; Fn, female neutered; LCP, locking compression plate; m, months; M, male; Mn, male neutered; PAUL, proximal abducting ulnar osteotomy; SSI, surgical site infection; w, weeks; y, years.

**FIGURE 1:**
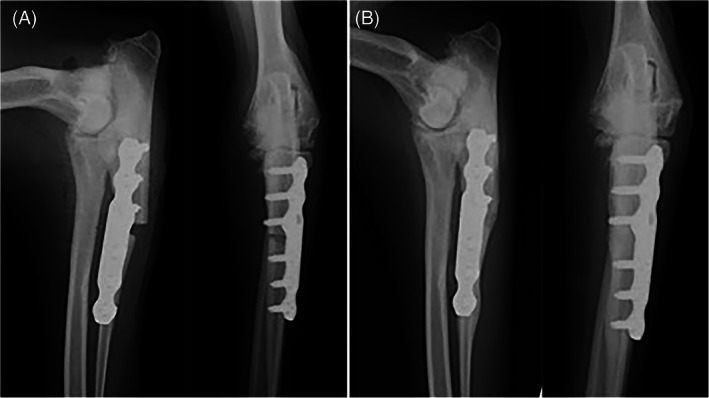
Immediate (A) and 12 week (B) post‐operative radiographs of a case where both experts predicted development of major mechanical complications, but no complications occurred. Both experts pointed out that the gap at the osteotomy site was excessive and there was inappropriate “caudal kick.” Additionally, Expert 2 also believed that the osteotomy site was too proximal and the plate was of an inappropriate size

**FIGURE 2:**
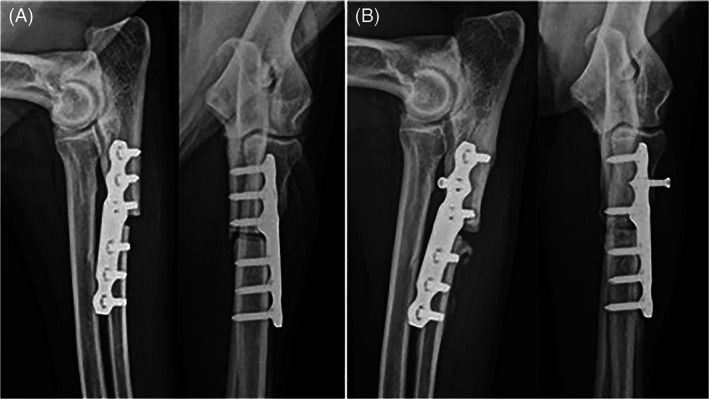
Immediate post‐operative radiographs (A) of case 70 where both experts predicted development of major mechanical complications, and complications subsequently occurred. In Expert 1 opinion, the plate was too oblique, there was excessive gap at the osteotomy site, and both plate and screws were of inappropriate size. In Expert 2 opinion, the plate was too oblique and too distal, the osteotomy was too proximal, there was an inappropriate “caudal kick,” and there was an excessive gap at the osteotomy site. At 12 weeks post‐operatively (B), the second screw was loose with migration, there was radiolucency around first and third screws, and there was an oligotrophic non‐union

**FIGURE 3:**
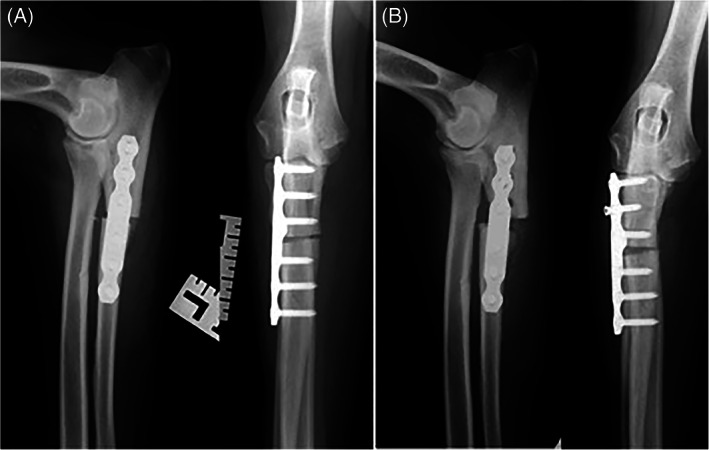
Immediate post‐operative (A) and 5‐week follow‐up (B) radiographs of case 7 where both experts predicted that there would be no major mechanical complications, but complications subsequently occurred. At 5 weeks post‐operatively, there was radiolucency around the first and third proximal screws, the second screw was loose with migration, and there was a delayed union of the osteotomy site

**FIGURE 4:**
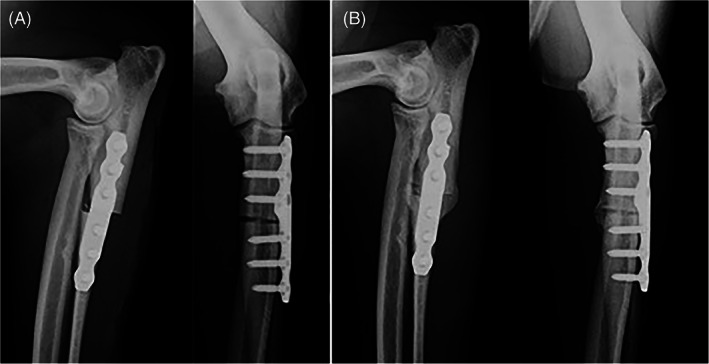
Immediate post‐operative (A) and 6‐week follow‐up (B) radiographs of a case where both experts predicted no development of major mechanical complications, and no complications were identified

Six of 19 limbs had a minor complication. Minor complications included ulnar fractures at the level of the empty hole left by the temporary 2.7 mm screw that was placed intra‐operatively (without nullifying the abducting effect of the PAUL plate) or across the most proximal locking screws (2), radiolucency around some of the locking screws (1), screw breakage (1), seroma (1), limited carpal extension/contracture of flexor tendons (1). Radiographs of case examples with and without complications are shown in Figures 1, 2, 3, 4.

Increasing *weight* (*p* = .01) was associated with the likelihood of complications and *being a Labrador* (*p* = .039) was associated with a reduced likelihood of complications occurring on univariate analysis. Age at the time of surgery, sex, and use of bandage post‐operatively were not associated with an increased risk of complications. An association between being young or old and development of complications could not be identified. Only *weight* was shown to be significantly associated with an increased risk of post‐operative complications (*p* = .04, OR 1.07, 95% CI 1.0–1.14) on multivariable logistic regression, equating to a 7% increase in risk for development of complications per kg of body weight. Being a Labrador was no longer associated with the likelihood of complications (*p* = .055) when *weight* was taken into account during multivariable logistic regression.

Expert 1 predicted the occurrence of complications in 16/74 limbs while Expert 2 predicted the occurrence of complications in 28/74 limbs. When asked “*Do you believe that these post‐operative radiographs show major technical errors that will lead to development of post‐operative complications*?” both experts showed low predictive ability (*k* = −0.08 for Expert 1 and *k* = 0.11 for Expert 2). Kappa values for Expert 1 and 2 when assessing the degree of agreement between “*implant errors*” and the likelihood of complications were −0.08 and 0.10 respectively. Kappa values for Expert 1 and 2 when assessing the degree of agreement between “*reduction errors*” and the likelihood of complications were −0.07 and 0.13 respectively. Expert 2 expressed “fair agreement” only for one category (*plate size [inappropriate]*; Kappa value = 0.23) when assessing agreement between specific subcategories of perceived technical error and development of complications. Kappa value was in the range −0.2 to 0.2 (slight agreement) for all radiographic factors considered in predicting complications for Expert 1, and for all other factors for Expert 2 (Tables 2 and 3).

**TABLE 2:** Two‐by‐two table and kappa values describing the degree of agreement between an overall assessment (and individual radiographic parameters) and the likelihood of complications for Expert 1

	Actual outcome		Kappa value^a^
	No complications	Complications	
Overall impression			
No complications	42	16	−0.08
Complications	13	3	
Plate positioning			
Acceptable	54	19	−0.03
Too cranial	1	0	
Acceptable	48	18	−0.09
Too caudal	7	1	
Acceptable	47	17	−0.05
Too oblique	8	2	
Acceptable	53	19	−0.05
Too proximal	2	0	
Acceptable	54	17	0.12
Too distal	1	2	
Acceptable	54	19	−0.03
Not in contact with bone	1	0	
Osteotomy positioning			
Acceptable	46	16	−0.007
Inappropriate caudal kick	9	3	
Acceptable	51	17	0.04
Excessive gap	4	2	
Acceptable	48	16	0.04
Too proximal	7	3	
Acceptable	54	19	−0.03
Too distal	1	0	
Screws			
Acceptable	51	17	0.04
Inappropriate length	4	2	
Acceptable	55	19	0
Inappropriate size	0	0	
Acceptable	49	17	−0.005
Not perpendicular to the plate	6	2	
Plate size			
Appropriate	50	18	−0.05
Inappropriate	5	1	
At least one implant error			
No	42	16	−0.08
Yes	13	3	
At least one reduction error			
No	43	16	−0.07
Yes	12	3	

^a^ Interpretation of *k*‐value: 0.01–0.20 slight; 0.21–0.40 fair; 0.41–0.60 moderate; 0.61–0.80 substantial; and 0.81–1.00 almost perfect. The value of −1.00 would indicate total disagreement and +1.00 would represent perfect agreement.

**TABLE 3:** Two‐by‐two table and kappa values describing the degree of agreement between an overall assessment (and individual radiographic parameters) and the likelihood of complications for Expert 2

	Actual outcome		Kappa value^a^
	No complications	Complications	
Overall impression			
No complications	36	10	0.11
Complications	19	9	
Plate positioning			
Acceptable	43	15	−0.008
Too cranial	12	4	
Acceptable	53	16	0.16
Too caudal	2	3	
Acceptable	49	14	0.18
Too oblique	6	5	
Acceptable	53	19	−0.05
Too proximal	2	0	
Acceptable	47	15	0.07
Too distal	8	4	
Acceptable	55	19	0
Not in contact with bone	0	0	
Osteotomy positioning			
Acceptable	42	13	0.08
Inappropriate caudal kick	13	6	
Acceptable	52	15	0.19
Excessive gap	3	4	
Acceptable	54	17	0.12
Too proximal	1	2	
Acceptable	48	15	0.10
Too distal	7	4	
Screws			
Acceptable	54	17	0.12
Inappropriate length	1	2	
Acceptable	54	16	0.19
Inappropriate size	1	3	
Acceptable	51	17	0.04
Not perpendicular to the plate	4	2	
Plate size			
Appropriate	51	14	0.23
Inappropriate	4	5	
At least one implant error			
No	38	11	0.10
Yes	17	8	
At least one reduction error			
No	37	10	0.13
Yes	18	9	

^a^ Interpretation of *k*‐value: 0.01–0.20 slight; 0.21–0.40 fair; 0.41–0.60 moderate; 0.61–0.80 substantial; 0.81–1.00 almost perfect. The value of −1.00 would indicate total disagreement and +1.00 would represent perfect agreement.

No agreement was reached (*k* = 0.0) when combining Expert 1 and 2 overall scores and assessing their predictive ability to the actual development of complications. Statistical power was too low to be able to evaluate whether combinations of specific subcategories of perceived technical errors might be associated with development of complications other than when grouped into “reduction errors” and “implant errors,” either for individual expert scores or combined expert scores.

Experts agreed that no complications were expected in 38 PAULs and those complications would be expected in eight limbs. However, the kappa coefficient for inter‐observer agreement (*k* = 0.12) was consistent with only a slight agreement between these two experts. Kappa value was 0.03 for the “*implant errors*” category and 0.17 for the “*reduction errors*” category (slight agreement) (Table 4).

**TABLE 4:** Two‐by‐two table and kappa values describing agreement between Expert 1 and 2 for at least one “implant error” and “reduction error” categories

	Expert 2 assessment		Kappa value^a^
	No implant error	Implant error	
Expert 1 assessment			
No implant error	38	21	0.03
Implant error	9	6	

	No reduction error	Reduction error	
Expert 1 assessment			
No reduction error	41	17	0.17
Reduction error	8	8	

^a^ Interpretation of *k*‐value: 0.01–0.20 slight; 0.21–0.40 fair; 0.41–0.60 moderate; 0.61–0.80 substantial; and 0.81–1.00 almost perfect. The value of −1.00 would indicate total disagreement and +1.00 would represent perfect agreement.

## 4 DISCUSSION

In this study, we documented an overall complication incidence of 19/74 limbs (equating to approximately 25.6% of limbs) with 13/19 limbs being classed as major complications and we identified that increasing body weight was a risk factor for appearance of complications following PAUL. Our null hypothesis that surgeons cannot predict implant failure/development of mechanical complications simply based on post‐operative radiographic interpretation was confirmed. Our second hypothesis regarding the lack of agreement between surgeons in predicting development of mechanical complications was also confirmed.

To our knowledge, this is the first written study to describe complications and risk factors associated with PAUL in a large cohort of dogs. It is of note that we did not attempt to evaluate clinical outcome following PAUL but limited our study to documentation of the incidence and categorization of complications, along with their potential risk factors.

The overall rate of complications following PAUL appears relatively high (~25%) with almost all major complications related to mechanical failure or to osteotomy non‐union. Our complication rate appears lower than a similar study, presented as oral communication, regarding application of PAUL plate in 69 antebrachii where the reported complication rate was 42%^12^ although direct comparison with this study is not possible because of the different inclusion criteria and methodology applied and of the different categorization into major and minor complications. In our study, six dogs showed a lack of bone healing activity at the osteotomy site which is a higher incidence than that reported for other stabilized osteotomy procedures in dogs (e.g., tibial plateau leveling osteotomy, sliding humeral osteotomy) to the best of the authors' knowledge.^5^, ^8^, ^16^ In all of these limbs the most proximal screws were either broken or had signs of radiolucency around the shaft. This implies that the poor biologic activity experienced in those dogs may be attributable to excessive mechanical instability at the osteotomy site^13^, ^14^, ^15^, ^16^ although precise mechanism cannot be confirmed in these clinical cases, and it is conceivable that conversely, the implant failure may be subsequent to delayed osteotomy healing and therefore persistent instability at the osteotomy. It is worth noting that 10/74 elbows in this study were in dogs of less than 1 year old. While we did not identify the age at surgery as a statistically significant risk factor for development of complications, it seems reasonable to assume that incidence of osteotomy non‐union could be higher if only skeletally mature patients were being operated due to potentially lower biological bone activity in older patients). Performing PAUL only in skeletally mature patients is widely advocated by most surgeons and the implant manufacturers, so caution is warranted in comparing this aspect of our study with other dog populations. Performing PAUL in juvenile patients remains controversial and may even be contra‐indicated on clinical grounds, further discussion of which falls beyond the scope of this manuscript.

Development of osteotomy non‐union could also be due to iatrogenic thermal necrosis and reduced cellular activity at level of the osteotomy site. Thorough lavage was carried out while performing the osteotomy to try to limit this potential effect. Two relatively recent veterinary studies showed that the potential for bone thermal damage in tibial osteotomies never reached critical duration of damaging temperatures and that use of saline irrigation produced no significant effect on peak cutting temperatures.^17^, ^18^ Whether lavage is indicated during creation of the osteotomy during PAUL to limit thermal damage to the bone is a potential area for further study. Similarly, use of a sharp saw blade is widely advocated during performance of osteotomy procedures, with excessive blade wear from re‐use in multiple procedures having the potential to increase temperature at the cutting interface.^18^ Within our case series we did not record data regarding whether a new oscillating saw blade was used for each procedure, or whether saw blades were re‐used in some individuals, and given the clinical nature of the study, we did not make any attempt to measure temperature at the saw‐to‐bone interface intraoperatively. Whether re‐use of saw blades could be a factor in poor bone healing whilst performing PAUL is another possible area for future study. As a further consideration, we did not apply any autogenous bone graft or bone graft substitute to the osteotomy site at the primary surgery in this case series, in line with the previously recommended technique. Whether incorporation of bone grafting as a routine part of the surgical technique, or in select cases, might further reduce the incidence of osteotomy non‐union is unknown.

Similarly to our study, major complications reported in a recent study were persistent surgical site infection, implant failure/screw loosening, delayed union, and carpal flexor muscle contracture.^12^ The latter complication was reported in four limbs, three of which required surgical release of the flexor tendons. We experienced this kind of complication only in one limb (which was treated conservatively), and we think that this could be attributable to adhesions formation due excessively traumatic elevation of the soft tissue envelope at the level of the proximal ulna or to acute compartment syndrome due to a tight bandage or to excessive post‐operative bleeding or edema. This is a potential area for further study.

The rate of implant removal in this second study^12^ (15.4%) was similar to the one we experienced (11.5%); these rates still appear to be relatively high when compared to the rate of implant removal of other clean orthopedic procedures (2.6%–7.4%).^19^, ^20^, ^21^ If we were to consider only the limbs where the implants were removed due to persistent infection, this rate would drop in line with reported percentages for clean orthopedic procedures (2.7%) with implant removal in several dogs performed in order to facilitate surgical revision of osteotomy non‐union in our study.

Increased body weight would be expected to challenge the biomechanical properties of a plate‐screws construct and, consequently, the degree of stability at level of the ulnar osteotomy. Our study showed a significant association between weight and increased risk of post‐operative complications, with a 7% increased risk for every additional kilogram of weight. Moderate correlation between the increase in patient's weight and complication occurrence was also recently reported by a different study.^12^ The manufacturer of the PAUL implants has recently released new conical screws (KLS™ technology, Kyon, Boston, MA) that are reportedly 30% stronger and would be expected to make the entire construct stiffer; this change may help prevent instability at the osteotomy site which may reduce complication rate although further study would be required to confirm this. Body condition scoring would have been meaningful data to corroborate these findings but, unfortunately, this was not routinely recorded at our institution.

Breed predisposition (being a Labrador) appeared to be associated with a reduced likelihood of complications (OR 0.32) within the univariate analysis. Labrador Retrievers were confirmed to be a breed at risk for elbow dysplasia in a recent study analyzing breed predisposition for common orthopedic conditions^22^ and, in our study this breed was over‐represented. The same breed was over‐represented in a recent oral communication about complications following PAUL.^12^ One potential explanation for the results of the univariate analysis and the seemingly “protective” effect of being a Labrador is that this may be related to bone conformation or perhaps even surgeon familiarity with bone conformation in this breed, which may facilitate optimal surgery. Another potential explanation within our study population would be that the Labradors were typically of lower body weight compared to the other breeds that developed post‐operative complications in our study, and this was supported by the lack of statistical significance of this feature within our multi‐variate analysis.

Despite the fact that the two experts found several technical errors in the way the surgical technique was performed in a large number of limbs, their accuracy of predicting development of complications following PAUL was low. In fact their combined scores were higher at the time of identifying limbs that would not develop complications (*n* = 28) than for limbs that would actually develop complications (*n* = 3). The inter‐observer agreement of the surgeons who participated in this study was also considered to be “slight” based on low kappa values. This intimates that post‐operative radiographs are not helpful for predicting development of mechanical complications after PAUL, even when evaluated by experienced observers. The precise clinical implication of this is a potential subject for further study. For example, it is unknown whether immediate re‐operation based on subjective radiographic identification of a possible technical error should be considered or not as a means of reducing subsequent complication incidence. The relatively poor inter‐observer variability between the “Expert” observers within our study suggests that further work may be required to better define potential technical errors or imperfections. Our study only explored subjective identification of potential errors by our “expert” observers, so another area for future study might be to evaluate whether more objective or quantifiable parameters could be identified in this regard.

Our findings of poor association between radiographic evaluation and development of complications are in line with a recent study that confirmed that immediate post‐operative radiographs were poorly predictive of failure after distal humeral fracture repair, including when evaluated by experienced surgeons.^23^ A human study specifically analyzing post‐operative radiographic factors and patient‐reported outcome after total hip replacement also demonstrated that solely relying on the analysis of plain radiographs taken before discharge to identify the cause of complications may be inadequate.^24^ Similar conclusions were reached by a different study whilst investigating if post‐operative radiographs were needed or not following open reduction and internal fixation of mandibular fractures. In fact, whilst some patients developed worrying signs and symptoms despite the immediate post‐operative radiographs looking favorable (and needed revision surgery), other patients with unfavorable post‐operative radiographs did not develop complications.^25^

Our study is subject to several limitations including the relatively small and heterogeneous population of dogs involved, lack of availability of body condition scores within our clinical data, and factors such as widely varying durations of post‐operative bandaging, all of which might reduce the statistical power in attempting to determine factors predisposing to post‐operative complications. Several different surgeons with varying levels of experience were involved in the study and whilst it is not known how steep the learning curve for PAUL is, we need to acknowledge that this may have resulted in an increase number of post‐operative complications and the number of potential technical errors identified on post‐operative radiographs. However, all surgeons were experienced diplomate surgeons who were regularly performing other elbow region surgeries, osteotomy procedures, and surgeries involving application of plates and screws, and all had undergone formal training in PAUL as recommended by the implant manufacturers. It is also worth noting that our complication rate is in line with the results achieved in another large referral hospital^12^ with no other previously published studies documenting complication incidence following this procedure to the best of our knowledge. A final limitation is that the experts evaluating post‐operative radiographs were blinded to all clinical information regarding the dog or surgeon. This does not directly reflect clinical practice and focused their part of the study on radiographic appearance alone. It is also of note that we did not attempt to evaluate the clinical outcomes of dogs within this study following PAUL, which would be another area for future study.

In conclusion, PAUL carried a relatively high complication rate and this was associated with an increased body weight of the dog. Suboptimal plate and screws placement or osteotomy reduction on post‐operative radiographs were poorly predictive for the development of complications.

## CONFLICT OF INTEREST

Ingo Pfeil holds a US patent (patent grant number 10143504) on the PAUL apparatus and method.

## AUTHOR CONTRIBUTIONS

Alan Danielski: conception of study, study design, literature review, acquisition of data, data analysis and interpretation, drafting, revising, and approval of the submitted article; Alexander Krekis: literature review, acquisition of data, data analysis, revising and approval of submitted article; Russell Yeadon: study design, literature review, acquisition of data, data analysis and interpretation, drafting, revising, and approval of the submitted article; Miguel Angel Solano: data analysis and interpretation, approval of the submitted article; Tim Parkin: data analysis and interpretation, drafting, approval of the submitted article; Aldo Vezzoni: data analysis and interpretation, approval of the submitted article; Ingo Pfeil: data analysis and interpretation, approval of the submitted article.
